# Eosinophilic bronchiectasis increases length and cost of hospitalization: a retrospective analysis in a hospital of southern China from 2012 to 2020

**DOI:** 10.1186/s12890-024-02912-2

**Published:** 2024-02-26

**Authors:** Chengcheng Lei, Zhimin Zeng, Fengjia Chen, Yubiao Guo, Yangli Liu

**Affiliations:** 1 Department of Pulmonary and Critical Care Medicine, The First Affiliated Hospital of Sun Yat-Sen University, No. 58 Zhongshan 2nd Road, Guangzhou, Guangdong 510080 China; 2 Institute of Respiratory Diseases, Sun Yat-Sen University, No. 58 Zhongshan 2nd Road, Guangzhou, Guangdong 510080 China

**Keywords:** Bronchiectasis, Blood eosinophil count, Length of hospital stay (LOS), Hospitalization cost, Inflammation markers

## Abstract

**Background:**

The concept of eosinophilic bronchiectasis has received clinical attention recently, but the association between blood eosinophil count (BEC) and hospital characteristics has rarely been reported yet. We aim to investigate the clinical impact of BEC on patients with acute bronchiectasis exacerbation.

**Methods:**

A total of 1332 adult patients diagnosed with acute exacerbation of bronchiectasis from January 2012 to December 2020 were included in this retrospective study. A propensity-matched analysis was performed by matching age, sex and comorbidities in patients with high eosinophil count (≥ 300 cell/µL) and low eosinophil count (< 300 cell/µL). Clinical characteristics, length of hospital stay (LOS), hospitalization cost and inflammatory markers were compared between the two groups.

**Results:**

Eosinophilic bronchiectasis occurred in approximately 11.7% of all patients. 156 propensity score–matched pairs were identified with and without high eosinophil count. Eosinophilic bronchiectasis presented with a longer LOS [9.0 (6.0–12.5) vs. 5.0 (4.0–6.0) days, *p* < 0.0001] and more hospitalization cost [15,011(9,753–27,404) vs. 9,109(6,402–12,287) RMB, *p* < 0.0001] compared to those in non-eosinophilic bronchiectasis. The median white blood cell (WBC), lymphocyte, platelet (PLT) and C-reactive protein (CRP) levels in eosinophilic bronchiectasis were significantly increased. Multivariate logistic regression analysis confirmed that the high levels of eosinophil count (OR = 13.95, *p* < 0.0001), worse FEV1% predicted (OR = 7.80, *p* = 0.0003) and PLT (OR = 1.01, *p* = 0.035) were independent prognostic factors for length of hospital (LOS) greater than 7 days.

**Conclusion:**

Eosinophilic bronchiectasis patients had longer length of hospital stay and more hospitalization cost compared to those in non-eosinophilic bronchiectasis group, which might be associated with the stronger inflammatory reaction.

## Introduction

Bronchiectasis is a heterogeneous chronic pulmonary disease, characterized by irreversibly dilated airways and thickening bronchial walls but various etiologies and clinical symptoms [1–3]. Although scholars traditionally believed that activated neutrophils are essential in pathogenesis of bronchiectasis and neutrophilic inflammation could predict exacerbations and prognosis, the concept of eosinophilic bronchiectasis has been proposed recently and the clinical significance of blood eosinophil count (BEC) in bronchiectasis is being increasingly realized [4–7].

Previous studies have demonstrated that eosinophilic subtype in bronchiectasis exhibits different clinical traits [8, 9]. Bronchiectasis patients with high blood eosinophil cells are relevant to *Streptococcus*- and *Pseudomonas*-dominated microbiome profiles, and they are susceptible to exacerbate even though controlling the confounding effects of infection [10]. However, few research studies on clinical significance of BEC and its association with length and cost of hospitalization in acute bronchiectasis exacerbations.

In this study, we analyzed the proportion of high BEC in bronchiectasis patients in a hospital of southern China, and then compared clinical characteristic, length of hospital stay, hospitalization cost and inflammatory markers between eosinophilic and non-eosinophilic bronchiectasis to explore the clinical effect and mechanism of high BEC on exacerbation of bronchiectasis.

## Methods

### Study subjects

A retrospective cohort analysis was performed with adult patients who were checked for chest high-resolution computered tomography (HRCT) scanning and diagnosed as acute exacerbation of bronchiectasis in the First Affiliated Hospital of Sun Yat-Sen University from January 2012 to December 2020. The exacerbation was defined with following symptoms: increased sputum volume, deterioration of lung function, progressively worse dyspnea, recurrent hemoptysis or wheeze, and systemic upset [11]. All data was collected retrospectively according to the ethical standards of the observational research, so the requirement for written informed consent was waived, and the study protocol was approved by the Ethical Committee of Human Experimentation in the Sun Yat-Sen University (ID:2019213).

### Study design

**Fig. 1:**
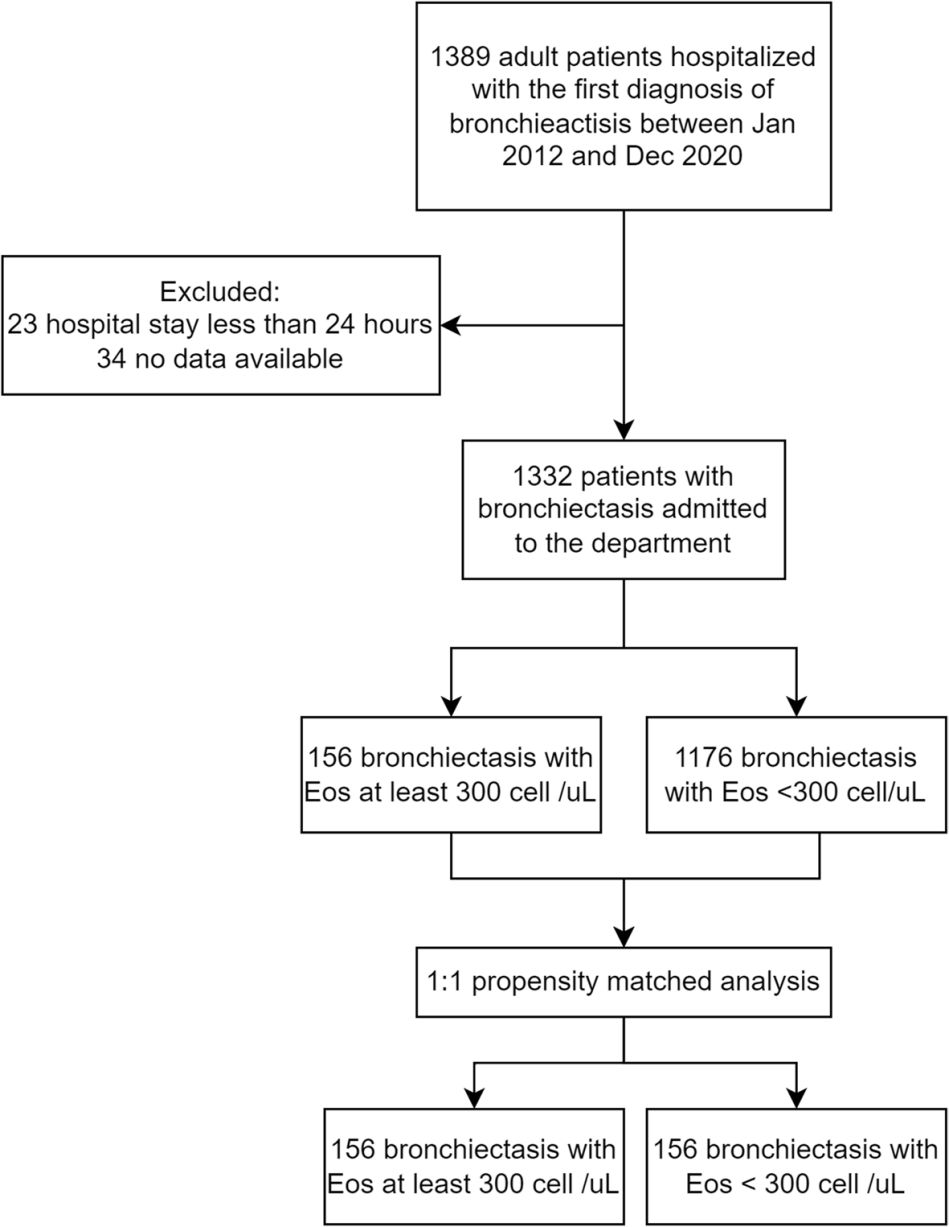
Flowchart of patient enrollment process. Abbreviations: EOS = Eosinophil count

The patient enrollment process is shown in Fig. 1. Blood and sputum samples were obtained at the beginning of the hospitalization before antibiotic treatment. According to peripheral blood cell count recorded on the first day of admission in the hospital, patients were divided into eosinophilic bronchiectasis group (Eos group) and non-eosinophilic bronchiectasis group (Non-Eos group). Specifically, patients’ absolute BEC ≥ 300 cell/µL at admission were defined as eosinophilic bronchiectasis [7, 10].

Demographic and clinical characteristics information of all patients were collected, including age, gender, body mass index, smoking status, length of hospital stay, medical costs, admission to ICU and history of asthma, COPD, diabetes, pulmonary tuberculosis, hematological malignancy, connective tissue disease, renal disease, congenital immunodeficiency, solid tumor, cardiovascular disease. Besides, data of pulmonary function test and systemic inflammatory indicators were retrieved from the hospital information system. Lung function test, mMRC score, and CT scan was assessed before discharge. The FACED score, which comprised of FEV_1_ predicted, age, *Pseudomonas aeruginosa* (*P. aeruginosa*) colonization, radiological extension and dyspnea, was calculated to evaluate disease severity [12].

### Statistical analysis

All data are presented as number for categorical variables, and median (interquartile range [IQR]) for non-normally distributed continuous variables. The baseline data of eosinophilic and non-eosinophilic bronchiectasis groups were analyzed using a Mann–Whitney U test for continuous variables and Pearson’s chi-square test for qualitative variables. A propensity score was developed for the exposure of blood eosinophil counts and matched eosinophil count ≥ 300 cell/µL to eosinophil count < 300 cell/µL. Propensity score matching (PSM) was performed based on 1-to-1 nearest neighbor matching with a caliper of 0.02 of the SD of the logit by matching the patients’ covariates: age, gender and comorbidities. Univariate logistic regression analysis with enter method was used to explore the effect of potential risk factors on length of hospital stay (LOS), and multivariate logistic regression analysis with stepwise selection was used to further assess independent factors. The statistical packages SPSS (version 25.0; SPSS, Chicago, IL, USA) and GraphPad Prism (version 8; GraphPad Software, San Diego, CA, USA) were applied for statistical analyses and drawing graphs, respectively. A two-sided value of *p* < 0.05 was considered statistically significant.

## Results

### Matched cohorts between eosinophilic bronchiectasis and non-eosinophilic bronchiectasis

Of 1,332 hospitalized bronchiectasis patients, 604 (45.3%) patients were male and the median age was 64 (53, 74) years old. The most common comorbidity disease was cardiovascular disease (43.8%), followed by COPD (13.7%), pulmonary tuberculosis (12.2%), diabetes mellitus (10.1%) and Asthma (5.6%). In the entire cohort, 156 (11.7%) patients’ BEC was ≥ 300 cell/µL. Compared with Non-Eos patients, the Eos group with BEC ≥ 300 cell/µL was more likely to suffer from asthma (9.6% vs. 5.0%, *p* = 0.018), COPD (20.5% vs. 12.8%, *p* = 0.008), and pulmonary tuberculosis (18.6% vs. 11.4%, *p* = 0.010). To verify the independent effect of eosinophilic cells on acute exacerbations, we conducted a PSM analysis with 1:1 ratio to generate 156 matched pairs for further analysis, in which the baseline characteristics were comparable and there was no difference between two groups in gender, age, and comorbidities after optimal matching (Table 1).

**Table 1:** Baseline characteristics of the entire cohort and propensity-matched sample

	Entire cohort			Propensity-matched sample		
	Non-Eos	Eos		Non-Eos	Eos	
	(*n* = 1176)	(*n* = 156)	*P*-value	(*n* = 156)	(*n* = 156)	*P-value*
**Clinical**						
Age,yrs	64[53, 74]	65[54, 76]	0.278	63[50, 73]	65[54, 76]	0.161
Male	522 (44.4)	82 (52.6)	0.054	68 (43.6)	82 (52.6)	0.113
**Years**			0.372			0.0002*
2012–2014	373(31.7)	55(35.3)		31(19.9)	55(35.3)	
2015–2017	414(35.2)	58(37.8)		48(30.8)	58(37.8)	
2018–2020	389(33.1)	43(26.9)		77(49.4)	43(26.9)	
**Comorbidities**						
Asthma	59(5.0)	15(9.6)	0.018*	16(10.3)	15(9.6)	0.850
COPD	150(12.8)	32(20.5)	0.008*	25(16.0)	32(20.5)	0.305
Diabetes mellitus	116(9.9)	18(11.5)	0.514	25(16.0)	18(11.5)	0.250
Pulmonary tuberculosis	134(11.4)	29(18.6)	0.010*	31(19.9)	29(18.6)	0.774
Hematological malignancy	11(0.9)	2(1.3)	0.679	3(1.9)	2(1.3)	0.652
Connective tissue disease	52(4.4)	3(1.9)	0.122	2(1.3)	3(1.9)	0.652
Renal disease	52(4.4)	10(6.4)	0.268	10(6.4)	10(6.4)	1.000
Congential immunodeficiency	4(0.3)	1(0.6)	0.564	0 (0)	1(0.6)	0.317
Solid tumor	144(12.2)	14(9.0)	0.235	10(6.4)	14(9.0)	0.395
Cardiovascular disease	510(43.4)	73(46.8)	0.417	63(40.4)	73(46.8)	0.254

Data are given as median [interquartile range] for coutinuous measures, and n (%) for categorical measures

*Abbreviations*: *COPD*   chronic obstructive pulmonary disease, *Eos* eosinophilic bronchiectasis, *Non-Eos*  non-eosinophilic bronchiectasis, *Statistically significant (*P* <0.05)

### Association between blood eosinophil cells and clinical features in bronchiectasis

The matched cohort included 156 patients with high eosinophil count (≥ 300 cell·µL^−1^) and 156 patients with low eosinophil count (< 300 cell·µL^−1^). Post pulmonary tuberculosis (19.2%), post-other infectious (22.1%), and chronic obstructive pulmonary disease (16.0%) were the most common etiologies. According to FACED score, 56.4% of patients were classified as mild disease severity, followed by moderate (33.3%) and severe (10.3%) in Eos group. After matching, patients in Eos group had higher median FVC% predicted (80% vs. 68%, *p* = 0.046) compared to those in Non-Eos group. However, no difference exists in other clinical characteristics including body mass index (BMI), smoking history, FEV1% predicted, FEV1/FVC ratio, age, mMRC dyspnea score, radiological severity, *Pseudomonas aeruginosa* and other pathogen colonization, exacerbation in the last year, treatment and FACED score between the two groups (Table 2).

**Table 2:** Clinical characteristics of subjects with eosinophilic bronchiectasis or non-eosinophilic bronchiectasis after propensity score matching

	Non-Eos	Eos	
	*N* = 156	*N* = 156	*P*-value
Body mass index, kg/m^2^	20.4 [18.3, 23.4]	20.8 [18.2, 24.0]	0.767
Smoking status, n (%)			
Never-smokers	116 (74.4)	114 (73.1)	0.726
Ex-smokers	25 (16.0)	27 (17.3)	0.658
Current Smokers	15 (9.6)	15 (9.6)	0.921
Microbiology, n(%)			0.115
P. aeruginosa	13 (8.3)	18 (11.5)	
H. influenzae	0 (0)	1 (0.6)	
NTM or TB	11 (7.1)	3 (1.9)	
Aspergillus	6 (3.8)	4 (2.6)	
Others	126 (80.8)	130 (83.3)	
Etiology, n (%)			0.891
Post pulmonary tuberculosis	31 (19.9)	29 (18.6)	
Post other infective	43 (27.5)	36 (23.1)	
COPD	23 (14.7)	27 (17.3)	
Connective tissue disease	2 (1.3)	3 (1.9)	
Immunodeficiency	0 (0)	1 (0.6)	
Others	7 (4.5)	10 (6.4)	
Unknown	50 (32.1)	50 (32.1)	
Lung function			
FEV_1_, % predicted	56 [39, 79]	69 [45, 89]	0.071
FVC, % predicted	68 [56, 87]	80 [64, 91]	0.046
FEV1/FVC ratio	70 [52, 79]	72 [57, 81]	0.35
FACED score			0.131
Mild (0–2)	98 (62.8)	88 (56.4)	
Moderate (3–4)	51 (32.7)	52 (33.3)	
Severe (5–7)	7 (4.5)	16 (10.3)	
Age years			0.098
≥ 70 years	49 (31.4)	63 (40.4)	
< 70 years	107 (68.6)	93 (59.6)	
FEV1% predicted			0.077
< 50%	33 (21.2)	31 (14.7)	
≥ 50%	122 (22.4)	123 (37.2)	
Chronic colonization by *P. aeruginosa*	13 (8.3)	18 (11.5)	0.344
mMRC dyspnoea score			0.515
Score I-II	136 (87.2)	132 (84.6)	
Score III-IV	20 (12.8)	24 (15.4)	
Lobes affected			0.21
1-2lobes	74 (47.4)	63 (40.4)	
> 2 lobes	82 (52.6)	93 (59.6)	
Exacerbations in the last year			0.566
0–2	124 (79.5)	128 (82.1)	
≥ 3	32 (20.5)	28 (17.9)	
Treatment			0.486
ICS + LABA	7 (4.5)	6 (3.8)	
LABA + LAMA	2 (1.3)	0 (0)	
Long-term (> 2 wk) macrolides	0 (0)	0 (0)	

*Abbreviation*s: *Eos*  eosinophilic bronchiectasis, *Non-Eos*  non-eosinophilic bronchiectasis, *FEV1*  forced expiratory volume in one second, *FVC* forced vital capacity, *P. aerugonisa*  Pseudomonas aeruginosa, *H. influenzae *Haemophilus influenza, *NTM*  non-tuberculous mycobacteria, *TB*  tuberculosis, *COPD*  chronic obstructive pulmonary disease, *mMRC*  Medical Research Council scale, *ICS* inhaled corticosteroids, *LABA* long-acting beta-agonists, *LAMA* long-acting muscarinic antagonists

### Disease burden in eosinophilic bronchiectasis

**Fig. 2:**
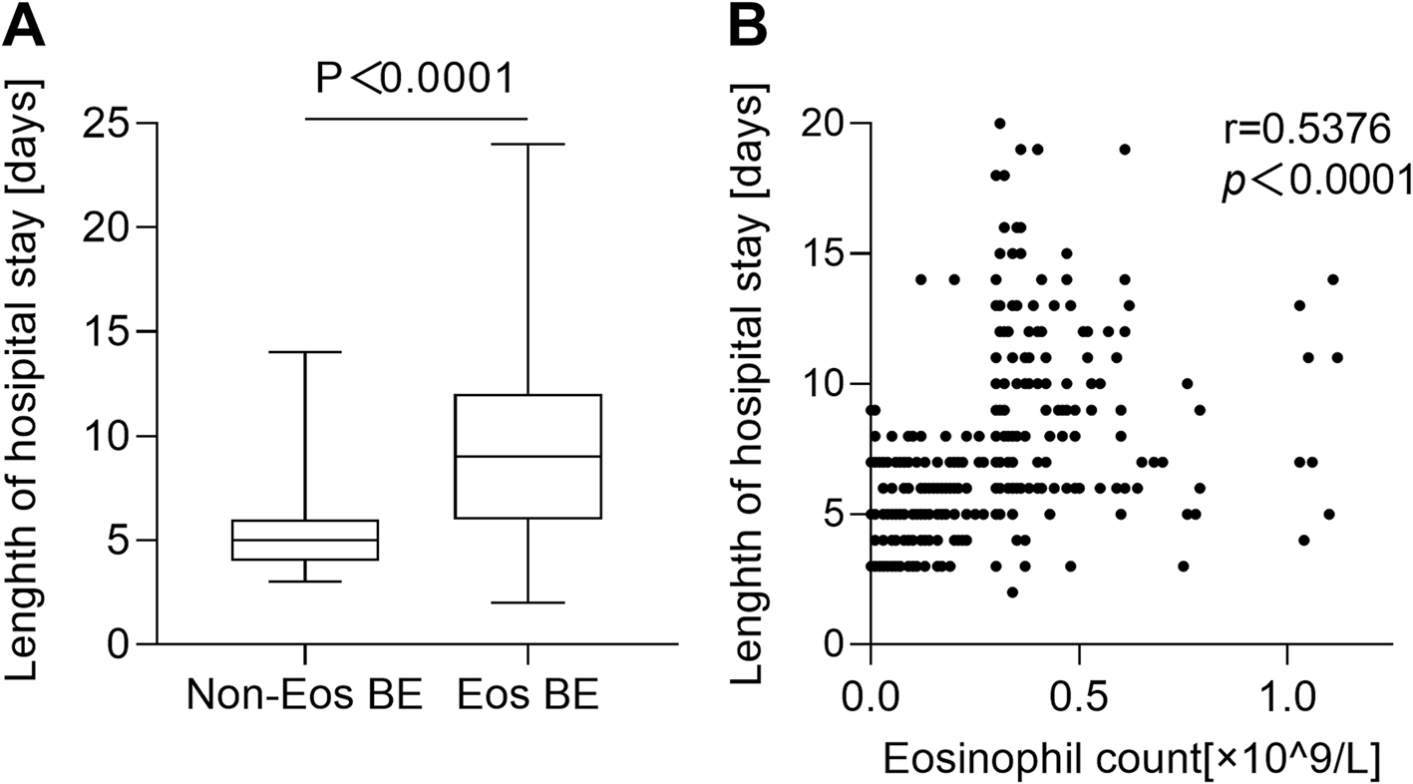
Length of hospital stay in patients with eosinophilic bronchiectasis and non-eosinophilic bronchiectasis (**A**) and the correlation between blood eosinophil count and length of hospital stay (**B**) after propensity score matching. Abbreviations: Non-Eos BE = non-eosinophilic bronchiectasis; Eos BE = eosinophilic bronchiectasis

As our results showed, the in-hospital medical cost for patients in Eos group was significantly higher than that in Non-Eos group [15011 (9753, 27404) vs. 9109 (6402, 12287) RMB, *p* < 0.0001]. Specifically, the Eos group cost more in medicine fee [5327 (2811, 9509) vs. 2187 (1059, 3650) RMB, *p* < 0.0001], and antibiotics fee [1995 (757, 4340) vs. 793 (434, 2271) RMB, *p* < 0.0001] (Table 3). Besides, the LOS of patients was significantly longer in Eos group [9.0 (6.0–12.5) vs. 5.0 (4.0–6.0) days, *p* < 0.0001] than that in Non-Eos group (Fig. 2A), which was positively correlated to BEC (*r* = 0.5376, *p* < 0.0001) (Fig. 2B). However, there was no statistically significance on the ICU rate (1.3% vs. 0%, *p* = 0.156) and respiratory failure (4.5% vs 7.1%, *p* = 0.331) in the two groups. No mortality was observed during hospitalization (Table 3).

**Table 3:** Medical costs, length of stay and clinical outcome of patients with eosinophilic bronchiectasis or non-eosinophilic bronchiectasis after propensity score matching

	Cost in RMB, Median (Range)		
	Non-Eos	Eos	
	(*n* = 156)	(*n* = 156)	*P*-vaule
Total cost	9109 [6402, 12287]	15011 [9753, 27404]	< 0.0001*
Medicine	2187 [1059, 3650]	5327 [2811, 9509]	< 0.0001*
Antibacterial	793 [434, 2271]	1995 [757, 4340]	< 0.0001*
Length of hospital stay	5.0 [4.0, 6.0]	9.0 [6.0, 12.5]	< 0.0001*
Admission to ICU, n (%)	0 (0)	2 (1.3)	0.156
Respiratory failure, n (%)	7 (4.5)	11 (7.1)	0.331
Mortality, n (%)	0 (0)	0 (0*)*	

*Abbreviations*: *ICU*  intensive care unit, *Eos* eosinophilic bronchiectasis, *Non-Eos* non-eosinophilic bronchiectasis

*Statistically significant (*P* < 0.05)

### Predictors for in-hospital stay of 7 days or more

In order to investigate the association between BEC and long LOS, univariate and multivariate logistic regression of long LOS (defined as ≥ 7 days) were performed with clinical variables. Univariate analysis results indicated that male, advanced age, worse lung function, high BEC, elevated CRP level, and raised PLT, WBC, neutrophil level were significantly associated with longer LOS. Further multivariate logistic regression analyses suggested that BEC ≥ 300 cells/μL (OR = 13.95, 95% CI 4.87–39.99, *p* < 0.0001), FEV1% predicted < 50% (OR = 7.80, 95% CI 2.60–23.43, *p* = 0.0003) and PLT (OR = 1.01, 95% CI 1.00–1.01, *p* = 0.035) predicted longer hospital stay for bronchiectasis patients (Table 4).

**Table 4:** Independent predictors of length of hospital stay of 7 days or more

	Univariate analysis		Multivariate analysis	
Parameter	OR (95% CI)	*P*-value	OR (95% CI)	*P*-value
Male	1.80 (1.15, 2.82)	0.01*		
Age (≥ 70 vs. < 70 years)	1.38 (1.09, 1.75)	0.007*		
FEV1% predicted (< 50% vs. ≥ 50%)	2.37 (1.23, 4.54)	0.10*	7.80(2.60, 23.43)	0.0003*
Eosinophil count (≥ 300 vs. < 300 cells/μL)	10.07 (5.98, 16.95)	< 0.0001*	13.95(4.87, 39.99)	< 0.0001*
CRP (≥ 7.2 vs. < 7.2 mg/L)	3.92 (2.21, 6.95)	< 0.0001*		
WBC (× 109/L)	1.12 (1.04, 1.20)	0.002*		
PLT (× 109/L)	1.01 (1.00, 1.01)	0.0002*	1.01(1.00, 1.01)	0.035*
Neutrophil count (× 109/L)	1.10 (1.02, 1.19)	0.013*		
Lymphocyte count (× 109/L)	0.96 (0.78, 1.17)	0.672		
Colonization of P. aeruginosa	1.59 (0.75, 3.36)	0.229		

The model uses logistic regression with length of stay stratified above and below 7 days

*Abbreviation*: *WBC*  white blood cell, *PLT*  platelet, *CRP* C-reactive protein, *CAR* C-reactive protein/albumin ratio, *P. aeruginosa*  pseudomonas aereginosa

*Statistically significant (*P *< 0.05)

### Inflammation markers in eosinophilic bronchiectasis patients

**Fig. 3:**
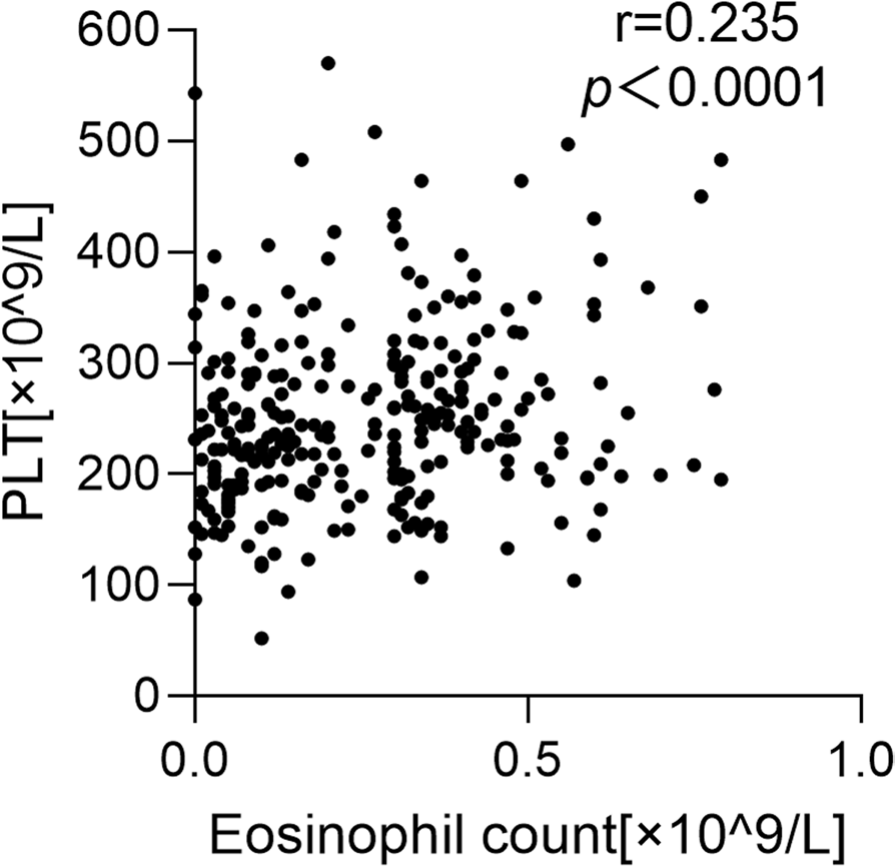
The correlation between blood eosinophil count and inflammatory marker after propensity score matching

Aiming to explore the underlying mechanism of eosinophilic bronchiectasis, we analyzed systemic inflammatory indexes in Eos group and Non-Eos group. The levels of WBC count [8.07 (6.63–9.27) vs. 6.76 (5.31–8.40), *p* = 0.021], lymphocyte count [1.78 (1.27–2.40) vs. 1.68 (1.23–2.25), *p* = 0.021], PLT count [258 (196–320) vs. 228 (193–266), *p* = 0.0003], and CRP [7.33 (2.50–38.20) vs. 3.87 (2.00–17.13), *p* = 0.026] were significantly higher in Eos group than those in Non-Eos group (Table 5). Moreover, the level of BEC positively associated with the number of platelets in peripheral blood (*r* = 0.235, *p* < 0.0001) (Fig. 3). However, there is no difference in other inflammatory indicators, such as procalcitonin, fibrinogen, albumin and D-D polymerization (Table 5).

**Table 5:** Inflammatory markers

	Non-Eos	Eos	
	(*n* = 156)	(*n* = 156)	*P*-value
WBC (× 10^9^/L)	6.76 [5.31, 8.40]	8.07 [6.63, 9.27]	0.021*
Lymphocyte (× 10^9^/L)	1.68 [1.23, 2.25]	1.78 [1.27, 2.40]	0.021*
Neutrophil (× 10^9^/L)	4.62 [3.54, 6.66]	4.78 [3.47, 6.65]	0.833
Hemoglobin (g/L)	128 [115, 134]	127 [115, 142]	0.107
PLT (× 10^9^/L)	228 [193, 266]	258 [196, 320]	0.0003*
CRP (mg/L)	3.87 [2.00, 17.13]	7.33 [2.50, 38.20]	0.026*
Procalcitonin (ng/L)	0.05 [0.04, 0.07]	0.05 [0.04, 0.10]	0.138
Fibrinogen (g/L)	3.36 [2.47, 4.40]	3.14 [2.41, 4.71]	0.263
Albumin (g/L)	39 [34, 42]	37 [33, 41]	0.182
D-D polymerization (mg/L)	0.41 [0.24, 0.94]	0.51 [0.25, 1.18]	0.081

Median and IQR of inflammatory markers according to the blood eosinophils at the day of admission

*Abbreviations*: *Eos* eosinophilic bronchiectasis, *Non-Eos* non-eosinophilic bronchiectasis, *WBC* white blood cell, *PLT* platelet, *CRP* C-reactive protein

*Statistically significant (*P* < 0.05)

## Discussion

Bronchiectasis has for a long time been regarded as excessive neutrophilic airway inflammation [4, 5, 13]. Until recently, the concept of eosinophilic bronchiectasis has attracted the attention of pulmonary specialists [10, 14], but it is rarely known about the clinical characteristics and disease burden of eosinophilic exacerbation bronchiectasis. Here, we innovatively reported that 11.7% bronchiectasis patients’ blood eosinophil cells were more than 300 cells/μL in southern China and showed that eosinophilic bronchiectasis patients had longer length of hospital stay and more hospitalization cost compared to those in non-eosinophilic bronchiectasis group, which might be due to the stronger inflammatory reaction. We assessed the effect attributable to high eosinophil cells with PSM analysis, and thereby minimizing the number of confounding variables and enhancing the validity of our results.

It is easily accessible to use blood eosinophil cell as a modest biomarker of airway inflammatory diseases, which is closely relevant to bronchial and/or lung eosinophilia. An European multi-cohort study had discovered that 18% ~ 29.5% of bronchiectasis patients showed a predominant eosinophilic airway inflammation by analyzing BEC [10], while in this southern China study, eosinophilic subtype was less common, accounting for 11.7% of bronchiectasis participants, similar to another retrospective report from Beijing, China which reported the prevalence of 17.6% [15]. The cause for the lower incidence rate might be consistent with lower incidence of allergic disease in China [16, 17]. We also found that the most common comorbidity disease was cardiovascular disease, which was in line with a previous study [18].

As for clinical characteristics, previous studies have reported conflicting findings. One observational study showed that bronchiectasis patients with high eosinophil counts (≥ 100cells/μL) exhibit a mild disease with better clinical outcomes, lung function parameters and nutritional status [19]. However, another study reported opposite results that bronchiectasis patients with a T2-high endotype (defined by either BEC ≥ 300cells/μL or FeNO ≥ 25bpp) exhibit a more severe disease with high dyspnea score, low lung function and worse quality of life [7]. The conflicting results in these findings were largely attributed to heterogeneity of bronchiectasis and complex comorbidities. PSM analysis was particularly useful for retrospective clinical trials to erase differences in baseline covariates between groups [20]. Our study controlled for the baseline factors including age, sex and comorbidities to evaluate the effect of eosinophil cells alone on the clinical outcomes and medical burden of bronchiectasis. Our results showed that no difference existed in clinical characteristics including BMI, smoking history, FEV1% predicted, FEV1/FVC ratio, dyspnea score, radiological severity and FACED score between the two groups, expect for higher FVC% predicted, which was in accordance with a multiple international cohorts study presenting that there is no significant differences exist in age, BMI, symptoms and FEV1 at baseline characteristics between eosinophilic bronchiectasis (defined by BEC ≥ 300cells/μL) and non-eosinophilic bronchiectasis after controlling for the confounding effects of infection [10].

The economic burden of acute exacerbation of bronchiectasis has been reported in a few of studies. The cost of one episode of bronchiectasis exacerbation was reported to be USD 7,827 in the United States [21] and EUR 5,284 in Spain [22], and patients with older age, more comorbidities, lower FEV1, chronic bronchial infection due to *P. aeruginosa*, and an association with COPD tended to be associated with higher healthcare costs [23]. Here, we firstly evaluated the economic cost of acute exacerbation of eosinophilic bronchiectasis in China and found that the median cost was RMB 15,011 for eosinophilic bronchiectasis, over 1.5 times as much as that with non-eosinophilic bronchiectasis (RMB 9,109). Moreover, the estimated medical cost of the matched EOS group may have been inadvertently underestimated due to inflation, particularly when the proportion of cases within the past 3 years was relatively smaller, suggesting that the Eos group would cause greater medical burden. Most of the total expenditure corresponds to longer hospital stay and higher medicine fee, especially antibiotics fee. This result was consistent with previous findings in other chronic airway disease such as asthma-COPD overlop, severe asthma and COPD, in which eosinophilic patients used more respiratory medications [24].

After adjusting for confounders, high eosinophil count remained a significant adverse factor on LOS in bronchiectasis, contrary to that the LOS was significantly shorter in COPD patients experiencing an eosinophilic exacerbation [25]. It is speculated that a rapid response to steroid treatment result in the shorter LOS in eosinophilic COPD. However, since the concept of eosinophilic bronchiectasis had been proposed until recently, corticosteroids have not been routinely recommended in clinical practice, which possibly prolong the length of hospital stay for eosinophilic bronchiectasis. Further prospective studies of the effects of inhaled and systemic corticosteroids on eosinophilic bronchiectasis exacerbation should be performed to better understand the clinical benefits. Moreover, we found that PLT but not CRP was related to longer hospital stay, in accordance with report from EMBARC registry [26] that thrombocytosis, but not CRP was associated with greater disease severity and increased hospitalization at 1 year. However, we did not find any association between *P. aeruginosa* and hospital length of stay. It should be noted that most of the microbiological information in this study was obtained through traditional sputum culture instead of 16S or next generation sequencing (NGS), so the detection rate was relatively low, which limited the results of clinical analysis.

For comprehending the underlying mechanism for poorer clinical outcome in eosinophilic bronchiectasis, inflammatory mediators were evaluated in this study. Elevated levels of WBC and CRP have been previously proven to be associated with increased risk of major comorbidities in COPD [27]. Increasing evidence also showed that PLT and CRP be associated with disease severity, exacerbations and worse clinical outcomes in bronchiectasis [26]. In our cohort, eosinophilic bronchiectasis presented with increased level of WBC, lymphocyte count, PLT, and C-reactive protein (CRP) and the number of PLT was positively associated with the level of BEC, suggesting the presence of elevated inflammation in eosinophilic subtype. Thus, these results suggested that the activated eosinophil cells released pro-inflammatory mediators resulting in sustained tissue inflammation and damage, which may be evolved in the mechanism underlying poorer clinical outcome in patients with eosinophilic bronchiectasis.

There are some limitations in our study. Firstly, we used the retrospective data from a single hospital and therefore inevitable biases exist, which should be verified in prospective multi-center clinical trials. Secondly, due to the large time-span of reviewed cases, the treatments involving corticosteroids and antibiotics could only be extracted from database, which lead to instances of missing data. Thirdly, definition of eosinophilic bronchiectasis based on a single measurement of eosinophil count during exacerbation of bronchiectasis was limited. Fourthly, the acute exacerbation and mortality were not followed up after the discharge to evaluate the long-term outcome. Despite these limitations, this study indeed adds evidence to support eosinophilic bronchiectasis as a specific phenotype driving increased disease burden and thus more attention in corticosterios treatment during exacerbation is needed.

## Conclusion

In general, Our data firstly uncovered that eosinophilic bronchiectasis patients displayed more hospitalization cost, longer LOS and stronger inflammatory response. By describing the above clinical characteristics of eosinophilic bronchiectasis, this study helps to provide more evidence in eosinophil targeted therapy in bronchiectasis. There is a need to further confirm our results and explore the mechanism underlying the eosinophilic inflammation and the consequent potential treatment.

## Data Availability

The original data supported this study can be looked up in our hospital’s electronic medical record system, further inquiries can be directed to the first author. Datasets are not suitable to be deposited to publicly available repositories due to patient privacy.
